# In-Situ Laser Polishing Additive Manufactured AlSi10Mg: Effect of Laser Polishing Strategy on Surface Morphology, Roughness and Microhardness

**DOI:** 10.3390/ma14020393

**Published:** 2021-01-14

**Authors:** Jiantao Zhou, Xu Han, Hui Li, Sheng Liu, Shengnan Shen, Xin Zhou, Dongqi Zhang

**Affiliations:** 1 The Institute of Technological Sciences, Wuhan University, South Donghu Road, Wuchang District, Wuhan 430072, China; zhou_jiantao@whu.edu.cn (J.Z.); leo_han@whu.edu.cn (X.H.); shen_shengnan@whu.edu.cn (S.S.); zhang_dongqi@whu.edu.cn (D.Z.); 2 Shenzhen Institute of Wuhan University, Keyuan South Road, Nanshan District, Shenzhen 518057, China; 3 Key Laboratory of Transients in Hydraulic Machinery, Ministry of Education, School of Power and Mechanical Engineering, Wuhan University, South Donghu Road, Wuchang District, Wuhan 430072, China; 4 Science and Technology on Plasma Dynamics Laboratory, Air Force Engineering University, Changle East Road, Baqiao District, Xi’an 710038, China; dr_zhouxin@126.com

**Keywords:** additive manufacturing, laser polishing, surface roughness, microhardness

## Abstract

Laser polishing is a widely used technology to improve the surface quality of the products. However, the investigation on the physical mechanism is still lacking. In this paper, the established numerical transient model reveals the rough surface evolution mechanism during laser polishing. Mass transfer driven by Marangoni force, surface tension and gravity appears in the laser-induced molten pool so that the polished surface topography tends to be smoother. The AlSi10Mg samples fabricated by laser-based powder bed fusion were polished at different laser hatching spaces, passes and directions to gain insight into the variation of the surface morphologies, roughness and microhardness in this paper. The experimental results show that after laser polishing, the surface roughness of *Ra* and *Sa* of the upper surface can be reduced from 12.5 μm to 3.7 μm and from to 29.3 μm to 8.4 μm, respectively, due to sufficient wetting in the molten pool. The microhardness of the upper surface can be elevated from 112.3 HV to 176.9 HV under the combined influence of the grain refinement, elements distribution change and surface defects elimination. Better surface quality can be gained by decreasing the hatching space, increasing polishing pass or choosing apposite laser direction.

## 1. Introduction

Additive manufacturing technology (AM), also known as 3D printing technology, is an advanced material forming technology. This technology is completely different from traditional manufacturing technologies. Parts with high density and complicated structure can be quickly obtained using AM technology [1,2]. Laser-based powder bed fusion (L-PBF) is a widely used metal additive manufacturing technology using a high-energy-density laser to scan through the metal powder bed [3]. AlSi10Mg alloy is a typical AM material. Many numerical and experimental studies regarding L-PBF AlSi10Mg parts have been reported [4,5]. Although the L-PBF process offers many advantages compared to traditional techniques, many defects inevitably occur in the L-PBF parts, such as unexpected rough surfaces and unavoidable pore defects and crack defects [6,7]. The mechanical properties of AM parts are significantly influenced by the surface roughness and defects. For example, the shallow surface cracks can lead to low fatigue resistance of AM stainless steel parts [8]. The fatigue strength is extremely affected by the surface roughness of AM Ti–6Al–4V specimens [9]. Thus, improving the surface quality, such as eliminating surface and sub-surface defects and reducing surface roughness, is of great value. The post-processing of L-PBF products has obtained more attention aimed at decreasing the defects and obtaining smooth surface.

In recent years, laser polishing has been widely used to improve the surface quality for various metals [10,11,12]. Temmler et al. [13] selected four sets of experimental process parameters to investigate the effect of multi-steps laser polishing on the microstructural properties of tool steel H11. They found that the surface roughness and the carbon concentration could be significantly reduced after laser polishing. The density could also be improved. Zhou et al. [14] experimentally studied the laser polishing titanium alloys. The experimental results showed that the surface roughness could be decreased from 7.3 μm to approximately 0.6 μm. Chen et al. [15] found that the surface roughness of laser polished fused deposition modelling (FDM) Al/PLA composite specimens was greatly reduced from 5.64 μm to 0.32 μm. Several surface defects were eliminated. Avilés et al. [16] investigated the effect of the laser polishing on the high cycle fatigue (HCF) performance of AISI 1045 steel. Experimental results showed that higher HCF strength could be obtained after laser polishing.

Laser polishing is also a useful method to ameliorate AM products’ properties. Many studies on laser polished AM parts have been reported. Laser polished AM CoCr alloy samples were found to have higher corrosion resistance [17]. Chen et al. [18] found that the sub-surface microhardness of the laser polished surface of AM 316L could be increased from 1.82 GPa to 2.89 GPa. The corrosion resistance could also be improved. Li et al. [19] applied laser polishing to AM Inconel 718. The laser polished layer consisted of equiaxed grain and columnar grain in microstructure. The laser polishing was also employed to control the surface wettability of AM CoCr component [20]. The experimental study has attracted more attention than the numerical study in recent years.

The effect of laser polishing is affected by many factors, such as laser energy density and polishing strategy. In our previous study, the effect of laser energy density on the laser polished surface roughness was investigated [21]. In this paper, a numerical model was established to study the rough surface evolution mechanism and complex hydrodynamic behavior in the molten pool during laser polishing with consideration of the phase transitions, gravity, recoil pressure, surface tension and Marangoni effect. The simulated temperature change and velocity distribution of the polishing surface were also discussed in detail. The effects of polishing hatching space pass and direction on the surface morphology, roughness and microhardness of L-PBF AlSi10Mg were investigated based on experiments. The corresponding mechanisms were also expounded.

## 2. Mathematical Modeling

### 2.1. Physical Model and Assumptions

**Figure 1:**
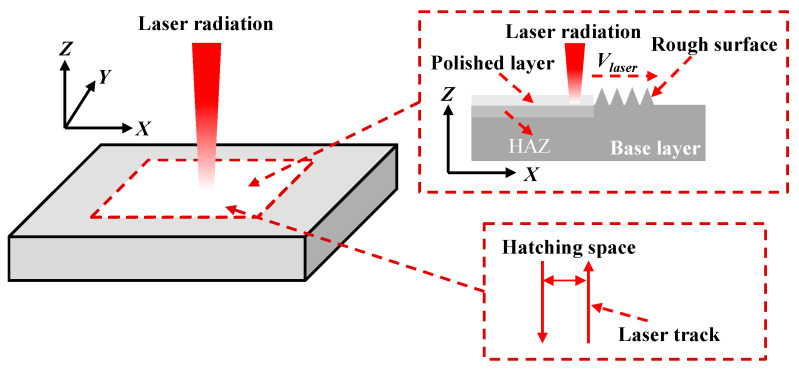
Schematic description of the laser polishing.

Nanosecond-laser polishing is based on the surface remelting to improve the surface quality of the part. A schematic description of laser polishing is shown in Figure 1. When the laser beam is irradiated on the upper surface, the temperature of the upper surface soon reaches its melting point, and then the molten pool and heated affected zone (HAZ) occur. The molten pool moves along the laser scanning direction.

**Figure 2:**
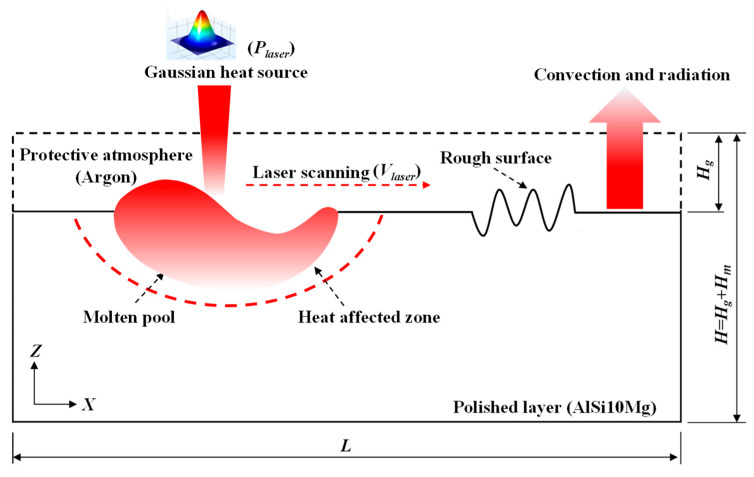
Representation of the model used in the numerical simulation.

To account for the rough surface evolution mechanism during laser polishing, the finite element method was used in the theoretical study. In this paper, the commercial software (COMSOL multi-physics 5.4, COMSOL Inc., Stockholm, Sweden) was employed considering heat transfer, laminar flow, gravity, recoil pressure, surface tension and Marangoni effect. The established time-dependent multi-physics coupled two-dimensional model was displayed in Figure 2. The level-set method was adopted to track the geometric properties of the interface between the rough surface and the protective atmospheric gas. As shown in Figure 2, the sizes of the laser polishing layer and protective argon gas zone were 2.5 mm × 1 mm and 2.5 × 0.5 mm, respectively. The upper surface was set as a random rough surface. The laser beam scanned along the positive *X*-axis. Both the heating and cooling time were 1 ms. The material physical properties and process parameters in this model are shown in Table 1.

To simplify the numerical calculations, several assumptions were proposed as follows:

(1) The laser beam is regarded as a continuous Gaussian heat source due to the short pulse separation pass of the laser (pulsed Nd: YAG laser) [27].
(2) The deformation of mechanical behavior has negligible effect on fluid field [27].
(3) The laser absorptivity of the material is assumed to be constant [27].
(4) The flow field in the molten pool is assumed as an incompressible Newtonian laminar flow [27].
(5) The material is isotropic and homogenous [27].
(6) The metal loss caused by evaporation during L-PBF is ignored [28].

### 2.2. Governing Equations and Boundary Conditions

As shown in Figure 2, the upper surface of laser polishing domain is irradiated by the laser heat flux. The convection and radiation to the environment were also considered. The heat transfer equation is governed by Fourier′s Law as follows:

(1) $$\rho C_{m}\left(\frac{\partial T}{\partial t}+u\cdot \nabla T\right)-\nabla \cdot (\lambda \nabla T)=Q,$$

where *ρ* is the density, *C_m_* is the material heat capacity, *λ* is the thermal conductivity, *Q* is the source term of heat transfer and *u* is the flow velocity.

The flow field in the molten pool can be calculated from the Navier–Stokes equation as follows:

(2) $$\rho \left(\frac{\partial u}{\partial t}+u\nabla u\right)=-\nabla \left[pI+\mu \left(\nabla u+{\left(\nabla u\right)}^{\tau }\right)\right]+F_{V},$$

where *p* is the flow pressure, *I* is the identity matrix, *μ* is the flow dynamic viscosity and *F_V_* is a source term corresponding to surface tension, gravity and Marangoni force.

The laser beam can be descripted as a Gaussian moving heat and the heat flux *Q_laser_* can be defined as:

(3) $$Q_{laser}=\frac{2AP_{laser}}{\pi r_{0}^{2}}\exp\left(\frac{-2[{\left(x-x_{0}-V_{laser}t\right)}^{2}}{r_{0}^{2}}\right),$$

where *A* is the laser beam absorptivity, *P_laser_* is the laser power, *r_0_* is the laser beam radius, *x_0_* is the initial laser beam position and *V_laser_* is the laser beam speed.

During laser polishing, when the temperature reaches the melting point of the material, the upper layer material starts to melt and transforms from the solid phase to the liquid phase. The heat capacity, *C_m_*, is used to describe the latent heat of phase change.

(4) $$C_{m}=\frac{\theta \rho_{s}C_{s}+\left(1-\theta \right)\rho_{l}C_{l}}{\theta \rho_{s}+\left(1-\theta \right)\rho_{l}}+L_{m}\frac{\partial \alpha_{m}}{\partial T},$$

where *C_s_* and *C_l_* are specific heat of solid and liquid, respectively. *L_m_* refers to the latent heat of fusion. *θ* is phase indicator, which can be described as:

(5) $$\theta =\{\begin{array}{c} 1\\ \left(T_{l}-T)/(T_{l}-T_{s}\right)\\ 0 \end{array}\begin{array}{c} T<T_{s}\\ \;\;\;\;\;\;\;\;\;\;T_{s}<T<T_{l}\;\;\;\;\\ T>T_{l} \end{array},$$

where *T_l_* is the liquid temperature and *T_s_* is the solid temperature.

*α_m_* is the latent heat distribution of phase change, which can be expressed as follows:

(6) $$\alpha_{m}=\frac{\left(1-\theta \right)\rho_{l}-\theta \rho_{s}}{2\left(\theta \rho_{s}+\left(1-\theta \right)\rho_{l}\right)},$$

where *ρ_s_* is the solid density and *ρ_l_* is the liquid density.

The Marangoni effect and the surface tension caused by the thermal gradient on the molten area can be given as follows:

(7) $$\sigma =\kappa \gamma \cdot n+\nabla_{s}\gamma \;,$$

where *κ* is the curvature, *γ* is the surface tension coefficient, **n** is the unit normal to the surface and ∇*_s_* is the surface gradient operator. ∇*_s_γ* represents the Marangoni effect due to the temperature gradient.

The escaping vapor leads to a recoil pressure as the temperature of the upper surface rises to the boiling point. The recoil pressure, *P_recoil_*, can be summarized as follows:

(8) $$P_{r\mathrm{ecoil}}=0.54P_{0}\exp\left(H_{V}\left(\frac{T-T_{v}}{RTT_{v}}\right)\right),$$

where *P*_0_ is the ambient pressure, *T* is the surface temperature, *H_V_* is the latent heat of evaporation, *R* is the universal gas constant and *T_v_* is the evaporation temperature.

### 2.3. Numerical Simulation Results and Discussions

**Figure 3:**
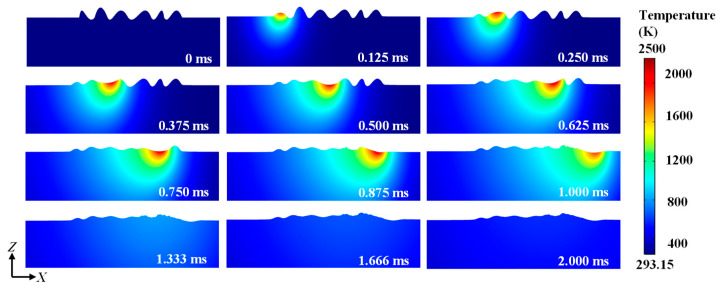
Temperature distribution during laser polishing.

**Figure 4:**
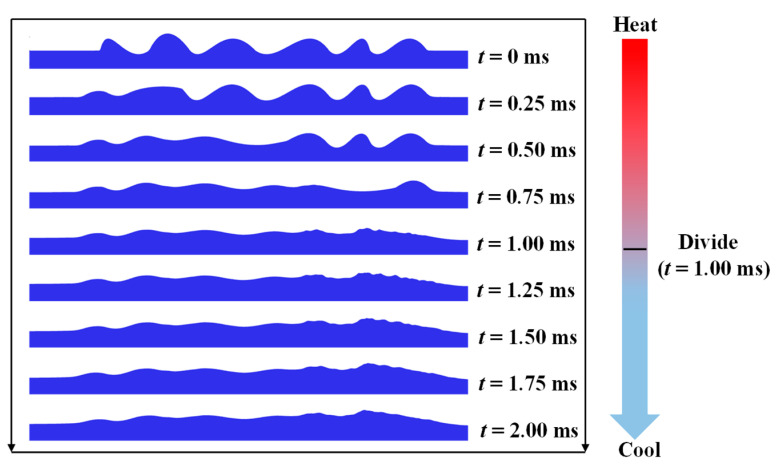
Evolution of the roughness during laser polishing.

The temperature distribution and the rough surface evolution process can be shown in Figure 3 and Figure 4. The temperature of the upper surface starts to rise under a stationary laser source. When the temperature rises to the melting point, the molten pool appears. The molten pool is gradually expanded upon continuous heating. It can be observed that there is a distribution of temperature gradient along the molten pool. The molten pool moves along the positive *X* direction as the laser beam moves. In the polished area, the material of the convex starts to flow to the concave at *t* = 0.25 ms. That is because the temperature coefficient is negative, so that the material tends to flow from high-temperature regions to low-temperature regions. At the same time, the height difference between the crest and trough is decreased gradually. At *t* = 1 ms, the upper surface starts to cool down. The high-temperature regions gradually disappear. Little change happens in the surface morphology due to the high cooling rate. After cooling (*t* = 2 ms), the free surface is obviously smoothed. However, due to the high heating and cooling rate, wetting and flow of the molten pool is not enough, so that a completely flat surface cannot be easily obtained.

**Figure 5:**
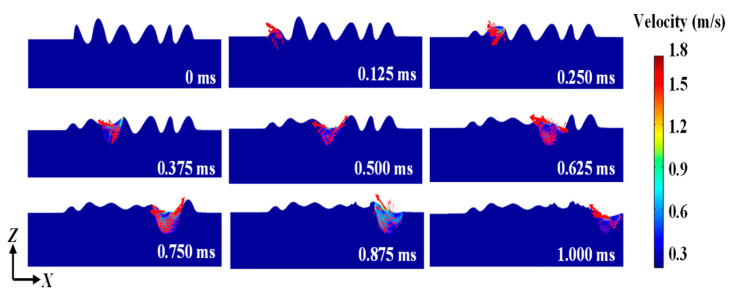
Velocity distribution during laser polishing.

The velocity distribution can be shown in Figure 5. It can be observed that the maximum velocity can reach 1.8 m/s in the molten pool during laser polishing. Li et al. [29] found that the surface tension and Marangoni flow played key roles in smoothing the surface during laser polishing Ti–6Al–4V. In this paper, due to Marangoni flow and surface tension, the rough surface moves into the tail region of the molten pool. The vortex is generated in the molten pool, which had a great impact on the mass transfer and surface topography. Then, the molten material flows down under the effect of gravity. Up to 1 ms, the velocity of the flow in the molten pool approaches around zero. This can explain why the morphology does not change much in the cooling process.

## 3. Experimental Details

### 3.1. Sample Fabrication

**Figure 6:**
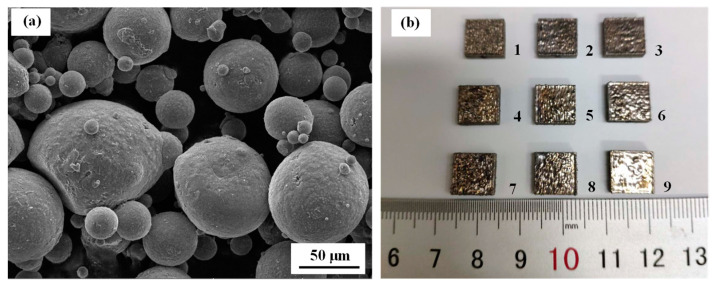
(**a**) SEM images of the AlSi10Mg powders and (**b**) representations of the samples.

The material of the powder particles was selected to be AlSi10Mg. Figure 6a shows scanning electron microscope (SEM, Tescan Mira3 SEM, Tescan Ltd., Brno, Czech Republic) images of the gas atomized AlSi10Mg particles. Most of the powder particles were spherical in shape. The particle diameter of the AlSi10Mg metal powders used in this paper ranged from 20 µm to 60 µm. The average diameter of the powders was 30 μm. The nine samples, each measuring 10 mm × 10 mm × 2.5 mm, were fabricated using Dimetal-280 L-PBF equipment (South China University of Technology, Guangzhou, China). A continuous wave fiber laser (IPG YLR-400-WC, IPG Photonics Corporation, Oxford, MA, USA) with a wavelength of 1060 nm, an output power of 70 W and a spot size of 70 μm was used during the printing process. The laser scanning speed, hatching space and layer thickness were 1 m/s, 70 μm and 30 μm, respectively, in the manufacturing process. The cubic samples were printed at the center of the substrate plate.

**Figure 7:**
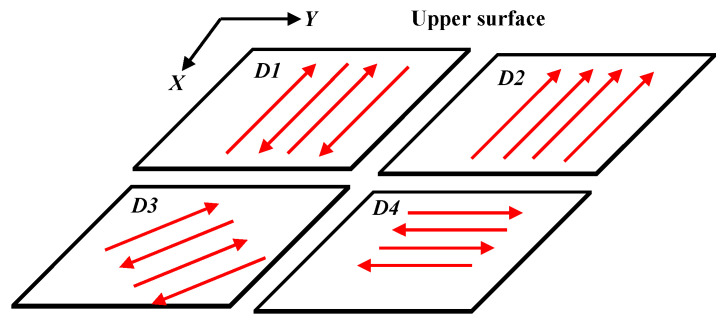
Schematic representations of different laser polishing directions.

After printing, there was no need to take the samples out of the printer. The laser of the printer was also employed to polish the samples. This enables in-situ polishing to avoid oxidation in the polishing process. The power and speed of the polishing laser were set to 400 W and 0.5 m/s, respectively, in this paper. The laser polishing strategies were listed in Table 2. The different polishing directions can be seen in Figure 7. The direction *D1* means that the polishing laser scans along as the same as the printing direction. The direction *D2* means that the polishing laser scans along negative *Y* direction. The directions *D3* and *D4* refer to the angles between the polishing laser and the printing laser as 45° and 90°, respectively.

After polishing, the samples were separated from the substrate by a wire cut, electric discharge machine (FH-020C, Suzhou Xingjie CNC Technology Co., Ltd., Suzhou, China) for follow-up measurement.

### 3.2. Morphology Observation, Roughness Tests, Cross-Section Observation and Microhardness Tests

**Figure 8:**
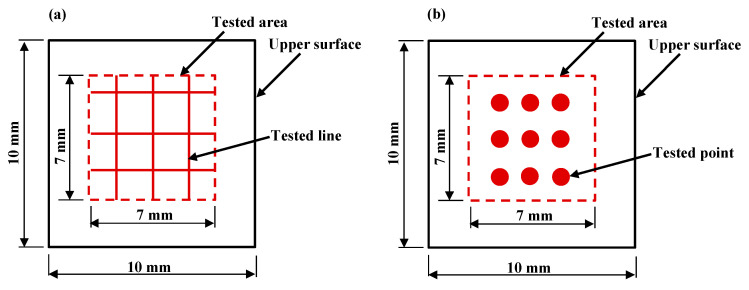
Schematic of tests: (**a**) *Ra* measurements and (**b**) microhardness tests.

The samples were ultrasonically cleaned in the alcohol for 20 min to remove residual powders and dirt. Scanning electron microscopy (SEM; Tescan Mira3 SEM, Tescan Ltd., Brno, Czech Republic) was employed to observe the microstructures. The energy dispersive spectrometer (EDS) was applied to take the elemental analysis. A comprehensive measurement system for surface profile (Form Talysurf PGI 830, Taylor Hobson Ltd., Leicester, UK) and 3D Optical Profiler (NewView 8000, ZYGO Ltd., Middlefield, OH, USA) were respectively taken to measure the surface roughness of *Ra* and *Sa*. *Ra* and *Sa* can be defined as follows. On the upper face of each sample, the six measurement results were averaged to obtain the final *Ra* value. The center zones of the samples were selected as the *Sa* measurement areas. The sizes of the upper surface and *Ra* tested area were 10 mm × 10 mm and 7 mm × 7 mm, respectively. The description of the *Ra* measurement is shown in Figure 8a.

(9) $$Ra=\frac{1}{l}\int_{0}^{l}\left|y(x)\right|dx$$

(10) $$Sa=\frac{1}{A}\iint_{A}\left|Z\left(x,y\right)\right|\;dxdy$$

where *l* is the surface profile length, *y*(*x*) is the deviation of the surface profile at the point *x* from the mean surface profile height, *A* is the measured area and *Z*(*x*, *y*) represents the height of the surface, relative to the best fitting surface.

The cross-sections of the samples with/without laser polishing were observed with an optical microscope (OM, MR-5000, Nanjing, China). Before observation, the cross-sections of the samples were etched by Keller′s solution (1%HF + 1.5% HCL + 2.5%HNO3 + 95%H_2_O).

The Vickers microhardness measurement of the upper surfaces of the samples was carried out on a Vickers microhardness testing machine (430SVD, Wilson Ltd., Fort Wort, TX, USA). A load of 9.8 N and an indentation pass of 10 s were used in the Vickers microhardness tests. As can be seen in Figure 8b, the nine tested points around the center of the upper surface were chosen to measure. The final microhardness value was obtained by averaging the nine values.

### 3.3. Experimental Results and Discussions

**Figure 9:**
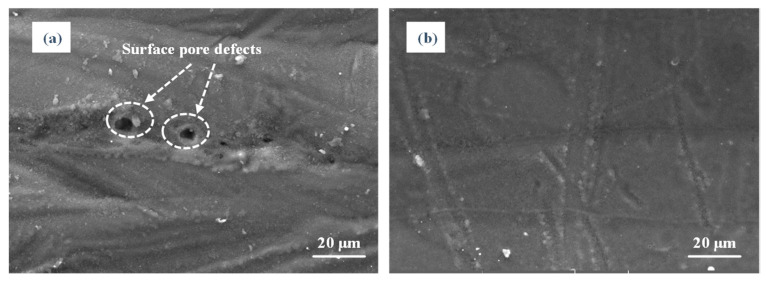
SEM images of the surface pore defects elimination caused by laser polishing: (**a**) unpolished surface and (**b**) polished surface.

**Figure 10:**
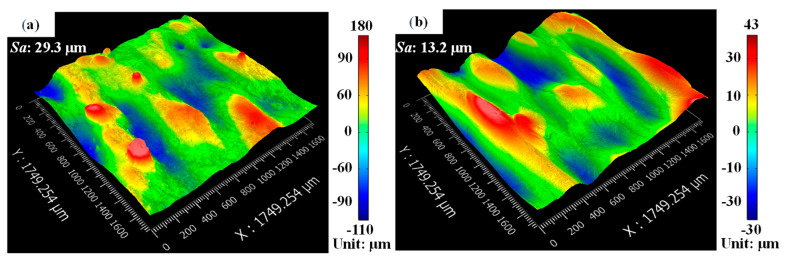
Optical morphologies of the surface: (**a**) unpolished surface and (**b**) polished surface of sample 2.

Figure 9 depicts that the surface pore defects can be eliminated by the post laser. Under the input of the polishing laser energy, molten material was transported along the temperature gradient [30]. Due to surface tension and Marangoni force, the surface pore defects were dragged to the tail in the molten pool, connected with the previous solidified layer, and finally removed. The optical morphologies of the surfaces with/without polishing can be shown in Figure 10. After laser polishing, there were more ordered and obvious tracks and less large peaks and valleys on the upper surface. The height of the peak and the depth of the valley were obviously reduced. The surface roughness of the nine samples are shown in Table 3.

**Figure 11:**
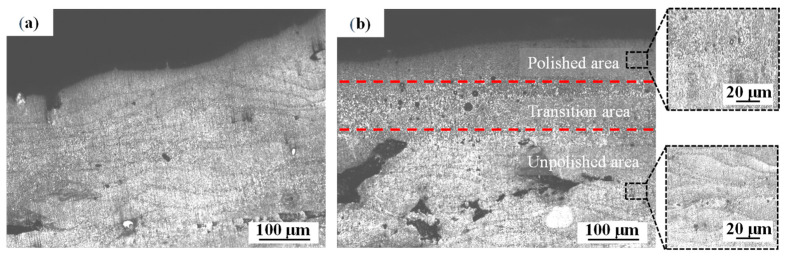
Optical images of the cross-section of (**a**) unpolished sample and (**b**) polished sample.

As shown in Figure 11, after polishing, the microstructure changed from a coarse columnar grain structure to fine equiaxed grain structure. The thermal undercooling caused by high cooling rates during laser polishing promoted the grain-refined effect [31]. Due to the Hall-Patch relationship, the finer grains caused by overlapping remelting can lead to the mechanical change.

#### 3.3.1. Effect of Hatching Space

**Figure 12:**
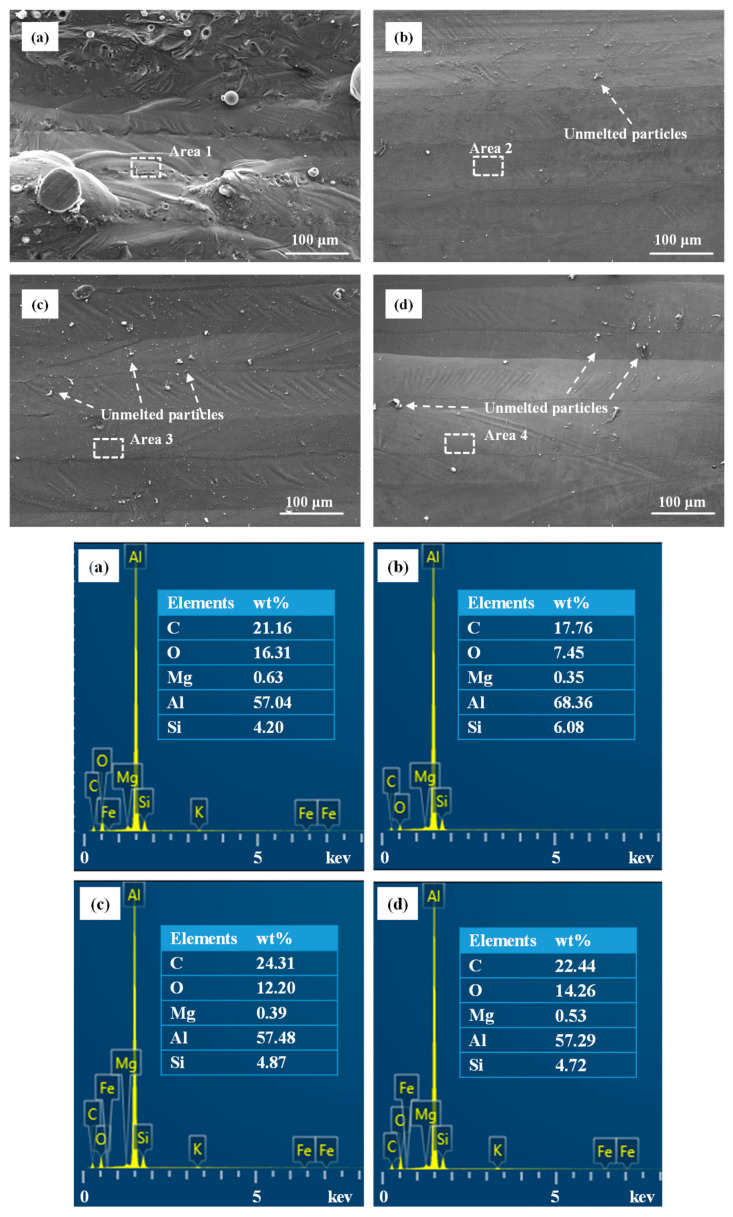
SEM images and EDS spectrums of the as-built and polished surfaces with different hatching spaces: (a) as-built, (**b**) *H_re_* = 40 μm, (**c**) *H_re_* = 70 μm and (**d**) *H_re_* = 100 μm.

The SEM images of the laser polished samples with different hatching spaces are shown in Figure 12. The EDS spectrums of the white area of the SEM images were also depicted. It can be observed that after laser polishing, the upper surface was significantly smoothed. The powder particles on the upper surfaces of the samples were remelted during laser polishing. The orderly melted tracks, a few unmelted particles adhered to the upper surface and several scratches could be clearly observed. At the hatching space of 40 μm, there were less unmelted powders on the upper surface. That is because that the hatching space of 40 μm is smaller than the laser spot diameter, which leads to remelting in some overlapping areas on the upper surface. Therefore, the generation of unmelted particles on the surface can be largely reduced.

**Figure 13:**
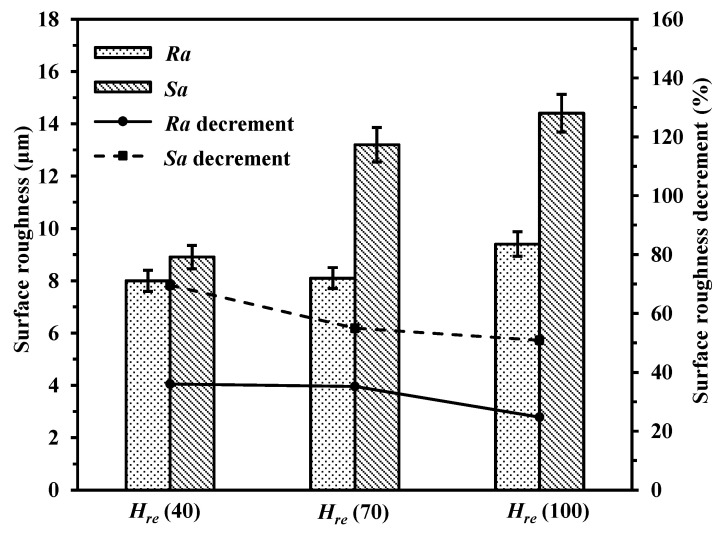
Surface roughness of polished surfaces with different hatching spaces (*H_re_* = 40 μm, 70 μm and 100 μm).

The surface roughness of the laser polished surfaces of the samples were evidently decreased compared with the as-built sample in Figure 13. The surface roughness of the *Ra* and *Sa* of the unpolished surface were 12.5 μm and 29.3 μm, respectively. *Ra* and *Sa* of the laser polished surfaces with different hatching spaces ranged from 7.2 μm to 9.4 μm and from 8.9 μm to 14.4 μm, respectively. At the hatching space of 100 μm, the unpolished area occurred due to the polishing tracks with gaps. Thus, the surface reduction was obviously less. It is the lower hatching space that can be chosen to reduce more surface roughness. Obeidi et al. [32] also found that increasing the overlapping area could improve the surface quality of AM 316L.

**Figure 14:**
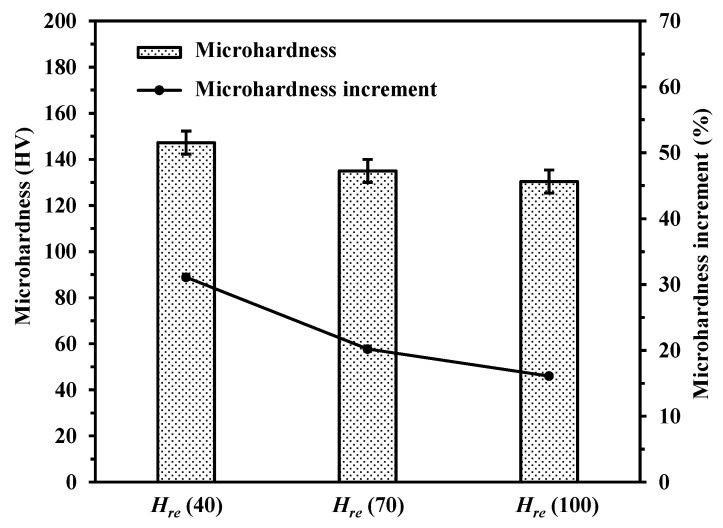
Microhardness of polished surfaces with different hatching spaces (*H_re_* = 40 μm, 70 μm and 100 μm).

As shown in Figure 14, the use of laser polishing resulted in significantly higher microhardness of the polished surfaces. The microhardness of the unpolished surface was 112.3 HV. The microhardness of the polished surfaces with different hatching spaces of 40 μm, 70 μm and 100 μm were 147.2 HV, 134.9 HV and 130.4 HV, respectively. The increments were 31.1%, 20.1% and 16.2%, respectively. Ipbal et al. [33] found that the existence of the defects had a great negative effect on the microhardness of AM 316L. As discussed above, the effect of surface defects elimination due to laser polishing is an important factor for improving microhardness. In addition, the solid-solution strengthening was also a factor that could not be ignored. In Figure 12, elements distribution was presented by EDS analysis. The Si and Mg elements play great roles in AlSi10Mg alloy mechanical properties [34]. The presence of Si and Mg elements in the samples can lead to solid-solution strengthening, which has a great effect on the mechanical properties [35]. EDS spectrums showed there were more Si and Mg elements on the polished surface with the hatching space of 40 μm, which may enhance the solid-solution strengthening effect.

#### 3.3.2. Effect of Polishing Pass

**Figure 15:**
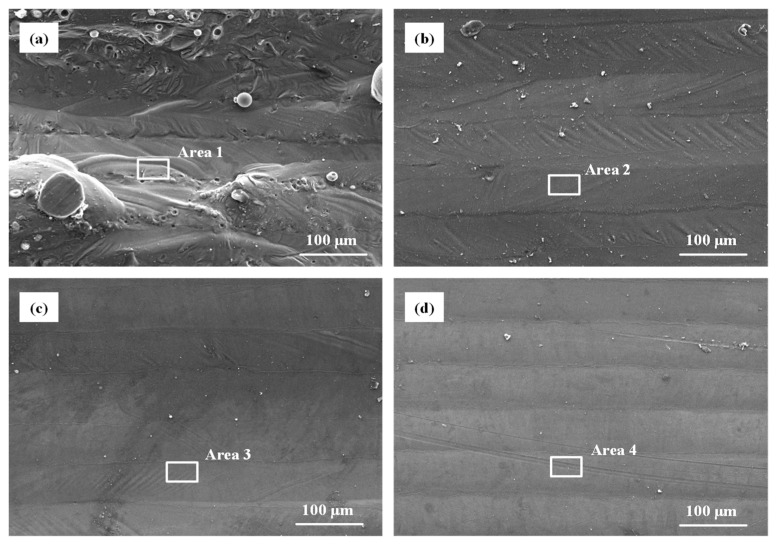

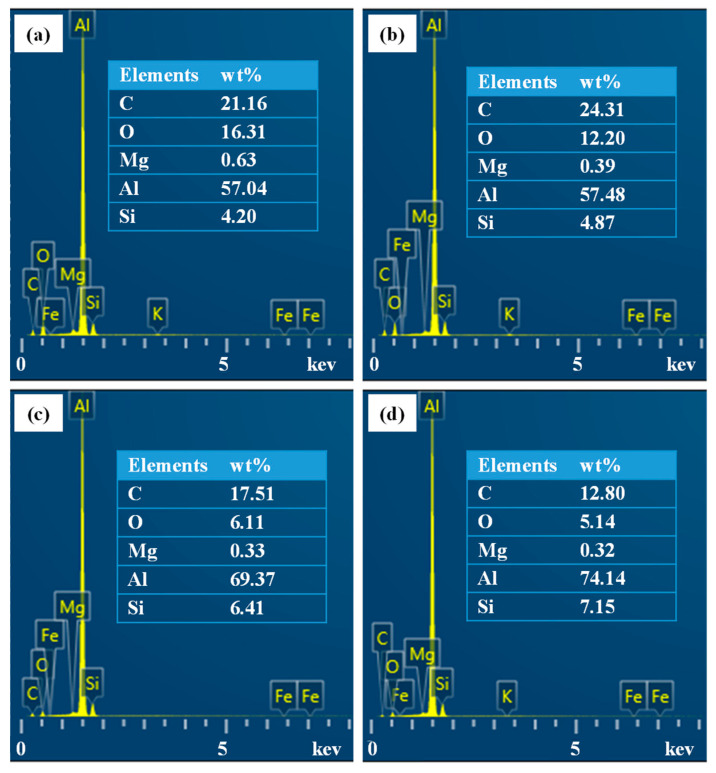
SEM images and EDS spectrums of the as-built and polished surfaces with different laser polishing passes (**a**) unpolished, (**b**) *L_t_* = 1, (**c**) *L_t_* = 2 and (**d**) *L_t_* = 3.

**Figure 16:**
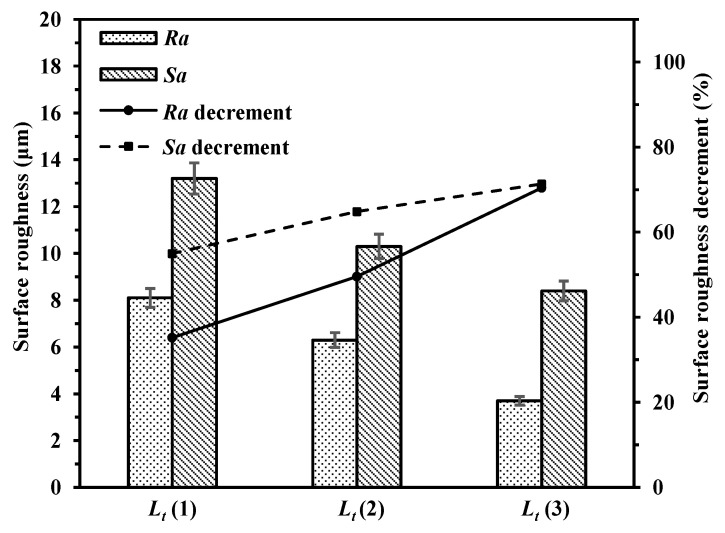
Surface roughness of polished surfaces with different laser polishing passes (*L_t_* = 1, 2 and 3).

SEM images and EDS spectrums of laser polished samples with different polishing passes are shown in Figure 15. After laser polishing according to the three passes strategy, there were more ordered molten tracks on the upper surface. Fewer unmelted particles were seen on the upper surface. As can be seen in Figure 16, *Ra* and *Sa* of the polished surfaces with different polishing passes of 1, 2 and 3 were ranged from 8.1 μm to 3.7 μm and from 13.2 μm to 8.4 μm, respectively.

**Figure 17:**
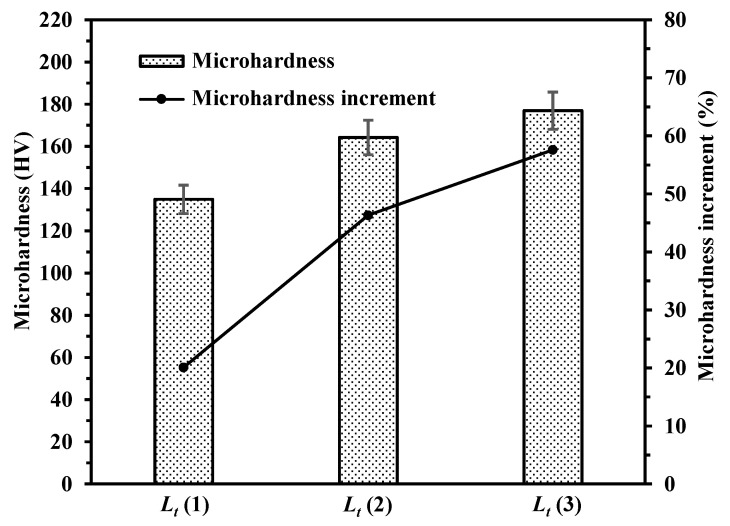
Microhardness of polished surfaces with different laser polishing passes (*L_t_* = 1, 2 and 3).

As shown in Figure 17, the microhardness of the laser polished surfaces with different polishing passes of 1, 2 and 3 were 134.9 HV, 164.3 HV and 176.9 HV, respectively. The increments were 20.1%, 46.4% and 57.6%, respectively. The repeated grain-refined effect and solid-solution strengthening enhance the microhardness increment. The next polishing laser can melt the remaining unmelted particles and rough surface, so that smaller roughness can be guaranteed. The surface can be smoothed by sufficient wetting during repeated polishing. The polishing effect was significantly improved with the increase of polishing passes.

#### 3.3.3. Effect of Polishing Direction

**Figure 18:**
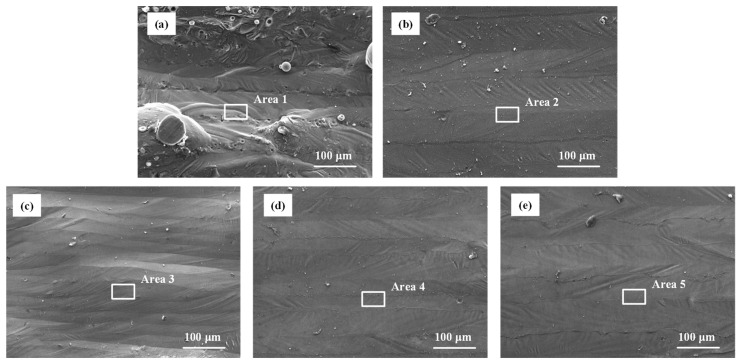

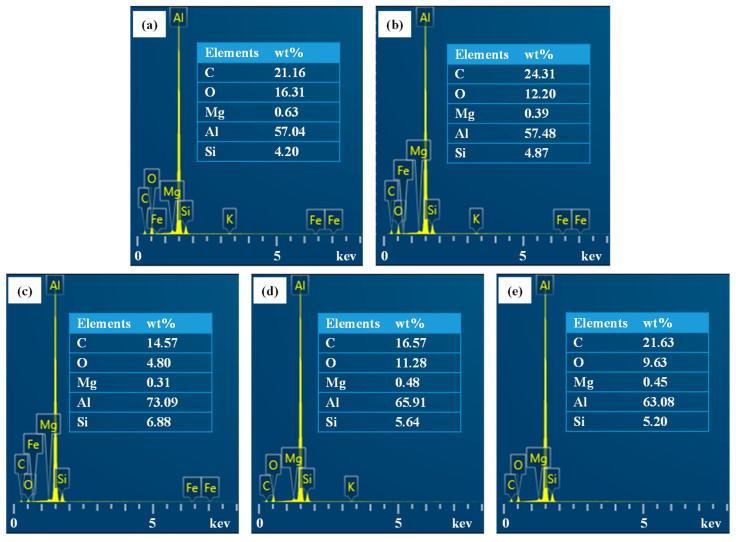
SEM images and EDS spectrums of the as-built and polished surfaces with different polishing directions: (**a**) unpolished, (**b**) *L_s_* = *D1*, (**c**) *L_s_* = *D2*, (**d**) *L_s_* = *D3* and (**e**) *L_s_* = *D4*.

The SEM images and EDS spectrums of the laser polished samples with different polishing directions are shown in Figure 18. It can be observed that the polishing direction is of great effect on the morphology of polished surfaces. There were fewer unmelted particles on the polished surface using the *D4* polishing direction. However, severe distorted tracks of the upper surface occurred because the wetting and diffusion process of the remolten area were affected by the remaining manufacturing laser tracks.

**Figure 19:**
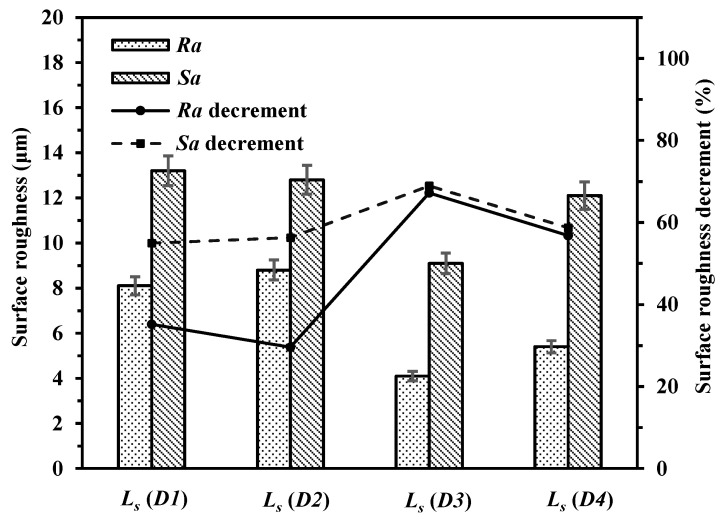
Surface roughness of polished surfaces with different polishing directions (*L_s_* = *D1*, *D2*, *D3* and *D4*).

**Figure 20:**
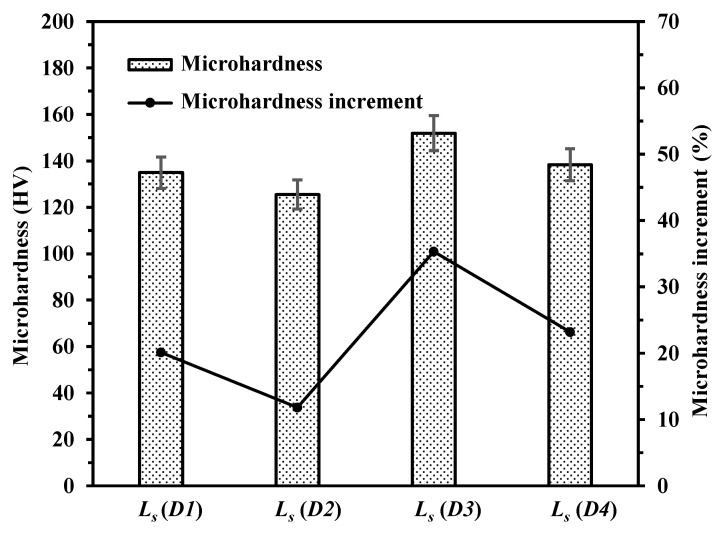
Microhardness of polished surfaces with different polishing directions (*L_s_* = *D1*, *D2*, *D3* and *D4*).

As can be observed in Figure 19, *Ra* and *Sa* of the laser polished surfaces with different polishing directions of *D1*, *D2*, *D3* and *D4* ranged from 8.8 μm to 4.1 μm and from 13.2 μm to 9.1 μm, respectively. In Figure 20, the microhardness of the laser polished surfaces with different polishing directions of *D1*, *D2*, *D3* and *D4* were 134.9 HV, 125.5 HV, 151.9 HV and 138.3 HV, respectively. The microhardness increments were 20.1%, 11.8%, 35.3% and 23.2%, respectively. At the laser polishing direction of *D3*, the polishing time is a little longer so that the more sufficient wetting and diffusion could be ensured. Thus, lower surface roughness can be obtained.

## 4. Conclusions

In this paper, the effects of in-situ laser polishing on L-PBF AlSi10Mg alloy using different polishing strategies were investigated. Further, the effects of laser polishing strategies on the other mechanical properties of the polished samples, including tensile property, creep performance and fatigue resistance, will be studied in our following study. The main conclusions can be summarized as follows:

(1) The laser polishing relies on the mass transfer caused by melting and re-solidification in the laser polishing process. The mass at the crests will move into the troughs due to sufficient wetting and diffusion of the molten pool. Thus, the rough surface can be significantly smoothed.
(2) The results of the experiment found clear support for the obvious effect of laser polishing. The surface roughness of the polished upper surface was greatly reduced and the microhardness was significantly increased after laser polishing. The surface roughness of *Ra* and *Sa* could be decreased by 70.4% and 71.3%, respectively. The microhardness could be increased by 57.6% after polishing.
(3) The hatching space, polishing pass and direction play great roles in the laser polishing effect. An appropriate polishing strategy can help to attain lower surface roughness, more ordered tracks and greater mechanical properties.

## Figures and Tables

**Table 1:** Material physical properties and numerical process parameters in the model.

Physical Property (Units)	Symbol	Value
Solid temperature (K)	*T_s_*	831.0 [22]
Liquid temperature (K)	*T_l_*	867.0 [22]
Evaporation temperature (K)	*T_v_*	2743.0 [22]
Base density of solid (kg·m^−3^)	*ρ_s_*	2690–0.19 (*T* = 293.15 K) [23]
Base density of liquid (kg·m^−3^)	*ρ_l_*	2482–0.27 (*T* = 846.15 K) [23]
Base thermal conductivity (W·m^−1^·K^−1^)	*λ*	113 + 1.06 × 10^−5^*T* [24]
Specific heat of solid (J·kg^−1^·K^−1^)	*C_s_*	536.2 + 0.035*T* [24]
Specific heat of liquid (J·kg^−1^·K^−1^)	*C_l_*	536.2 + 0.035*T* [24]
Dynamic viscosity of liquid (N·s/m^2^)	*μ*	1.3 × 10^−3^ [24]
Latent heat of fusion (J·kg^−1^)	*L_m_*	5.03 × 10^5^ [25]
Latent heat of evaporation (J·kg^−1^)	*H_V_*	1.07 × 10^7^ [25]
Emissivity	*ε*	0.36 [25]
Laser absorption coefficient	*A*	0.09 [25]
Convective coefficient (W·m^2^·K)	*h_c_*	80 [25]
Surface tension (N·m^−1^)	*σ*	(1000.72 − 0.152*T*) × 10^−3^ [26]
Specific heat of argon gas (J·kg^−1^·K^−1^)	*C_g_*	20.786 [27]
Base density of argon gas (kg·m^−3^)	*ρ_g_*	1.618 [27]
Dynamic viscosity of argon gas (N·s/m^2^)	*μ_Ar_*	22.676 × 10^−6^ [27]
Atmospheric pressure (Pa)	*P_a_*	1.01 × 10^−5^ [27]
Preheating temperature (K)	*T_0_*	293.15
Laser polishing power (W)	*P_laser_*	275
Laser radius (μm)	*r_0_*	50
Scanning speed (m·s^−1^)	*V_laser_*	0.5

**Table 2:** Laser polishing strategies in experiments.

Sample	Hatching Space (μm)	Polishing Direction	Polishing Pass
*No.*	*H_re_*	*L_s_*	*L_t_*
1	–	–	0
2	40	*D1*	1
3	70	*D1*	1
4	100	*D1*	1
5	70	*D1*	2
6	70	*D1*	3
7	70	*D2*	1
8	70	*D3*	1
9	70	*D4*	1

**Table 3:** Surface roughness of the samples.

Sample No.	Surface Roughness *Ra* (μm)	*Ra* Reduction (%)	Surface Roughness *Sa* (μm)	*Sa* Reduction (%)
1	12.5	–	29.3	–
2	7.2	42.4	8.9	69.6
3	8.1	35.2	13.2	54.9
4	9.4	24.8	14.4	50.9
5	6.3	49.6	10.3	64.8
6	3.7	70.4	8.4	71.3
7	8.8	29.6	12.8	56.3
8	4.1	67.2	9.1	68.9
9	5.4	56.8	12.1	58.7

## Data Availability

The data presented in this study are available on request from the corresponding author. The data are not publicly available due to the data also forms part of an ongoing study.
